# Paediatric Idiopathic Intracranial Hypertension (IIH)—A Review

**DOI:** 10.3390/life11070632

**Published:** 2021-06-29

**Authors:** Andrew Malem, Twishaa Sheth, Brinda Muthusamy

**Affiliations:** Paediatric Ophthalmology, Department of Ophthalmology, Cambridge University Hospitals NHS Foundation Trust, Cambridge CB2 0QQ, UK; Twishaa.sheth@addenbrookes.nhs.uk (T.S.); brinda.muthusamy@addenbrookes.nhs.uk (B.M.)

**Keywords:** headache, paediatric idiopathic intracranial hypertension, optical coherence tomography, papilloedema, pseudo tumour cerebti, vision, optic nerve

## Abstract

Paediatric idiopathic intracranial hypertension (IIH), is a rare but important differential diagnosis in children presenting with papilloedema. It is characterised by raised intracranial pressure in the absence of an identifiable secondary structural or systemic cause and is, therefore, a diagnosis of exclusion. In the adult population, there is a strong predilection for the disease to occur in female patients who are obese. This association is also seen in paediatric patients with IIH but primarily in the post-pubertal cohort. In younger pre-pubertal children, this is not the case, possibly reflecting a different underlying disease aetiology and pathogenesis. Untreated IIH in children can cause significant morbidity from sight loss, chronic headaches, and the psychological effects of ongoing regular hospital monitoring, interventions, and medication. The ultimate goal in the management of paediatric IIH is to protect the optic nerve from papilloedema-induced optic neuropathy and thus preserve vision, whilst reducing the morbidity from other symptoms of IIH, in particular chronic headaches. In this review, we will outline the typical work-up and diagnostic process for paediatric patients with suspected IIH and how we manage these patients.

## 1. Introduction

Paediatric idiopathic intracranial hypertension (IIH) is a rare condition affecting between 1 in 100,000–150,000 children in the population [1,2,3]. It is defined as raised intracranial pressure (ICP) in the absence of a site-occupying lesion, normal brain structure and vasculature, and normal metabolic, endocrine, and haematological systemic states. The revised diagnostic criterion for the diagnosis uses a lumbar puncture (LP) opening pressure of more than 28 cm H_2_O or more than 25 cm H_2_O if the child is not obese and not sedated [4].

The identification and diagnosis of IIH in children can be difficult. Limited examination compliance, variable clinical signs, and symptoms and the challenges associated with diagnostic investigations, which often require general anaesthesia or sedation, all add to this challenge. Despite these difficulties, accurate diagnosis is essential to limit long-term morbidity.

## 2. Demographics

Paediatric IIH prevalence increases with increasing age. Overall incidence rates of 0.9 cases by 100,000 are disproportionally spread across the paediatric age range with twice as many children aged 12–17 years old affected than 2–12 [5]. IIH is uncommon in infants and exceedingly rare in neonates [5,6]. A meta-analysis of the epidemiological characteristics of paediatric IIH was reported by Genizi et al. showed 44% of those affected under 12 years of age were female. This increased to 79% in the 12–17 age group [7]. Balcer et al. reported data from 40 patients demonstrating an equal split between males and females in the pre-pubertal group whilst the post-pubertal group was predominantly obese and female [8]. A more recent large multi-centre study by Sheldon et al. suggested the presence of three distinct age based subgroups: pre-pubertal, early adolescent, and late adolescent, each of which having different anthropometric characteristics [9]. Paediatric IIH shows no racial predilection, although it has been reported black patients with IIH have a higher risk of severe visual loss [10]. No genetic locus has been linked to IIH; despite this, familial cases have been reported [11].

## 3. Risk Factors

Commonly reported risk factors in adults affected by IIH include female sex, obesity, and being in the reproductive age range [4]. The female sex predilection seen in adult IIH patients is a risk factor for paediatric IIH, but only in older children and not in the younger pre-pubertal age group.

A well established association between obesity and IIH in adults exists [8]. A similar association is also present in paediatric IIH patients, with the prevalence of obesity increasing with age. In those under 12, rates of obesity are relatively low ranging from 26–43%. In post-pubertal children, obesity is present in 64–81% of 12–14-year-olds with IIH and 91% in those in the 15–17 age range [7,8]. More recently Sheldon et al. found three subgroups relating age with weight. Patient BMI Z scores in the youngest age group (under 7 years for boys and under 8.5 years for girls) were similar to age and height matched reference values. In the early adolescent subgroup, BMI Z values were clustered around overweight and obese levels. In the late adolescent group (over 12.5 years old for boys and girls) BMI Z-levels were well above the overweight and obese reference levels [9].

## 4. Pathophysiology

Idiopathic intracranial hypertension is a diagnosis of exclusion, made only when the presence of a structural brain or vascular lesion, and associated systemic pathology/medications have been excluded. If raised ICP is confirmed and an associated systemic pathology or medication use is present, the term pseudotumour cerebri syndrome is used. A variety of medications are known to be associated with pseudotumour cerebri syndrome which results in raised ICP through a variety of mechanisms [4]. For the purpose of this review, we will focus on the proposed mechanisms underlying IIH [12,13,14,15,16,17,18,19,20].

Elucidating the underlying disease mechanism behind IIH has remained somewhat elusive. Extensive research has been conducted on this question, although predominantly in adults. However, similarities between patient demographics and risk factors in the early and late adolescent post-pubertal paediatric groups and adult IIH, points towards a shared underlying disease mechanism. Likewise, the clear differences seen in the pre-pubertal paediatric IIH demographics suggest in this age group, IIH is likely due to a different underlying disease mechanism.

Different theories surrounding CSF fluid dynamics have been proposed including excessive CSF production, increased cerebral blood volume, and brain oedema. Most attention has however focused on decreased CSF drainage due to abnormalities in the arachnoid villi or venous circulation [5]. Decreased CSF drainage has been demonstrated with radioisotope cisternography [12]. However, it has been postulated the reduced flow through the arachnoid villi is secondary to the raised ICP causing compression of the villi as opposed to primary abnormalities in the arachnoid villi causing the elevated ICP [13].

In addition to the proposed mechanisms of reduced flow through the arachnoid villi, abnormal CSF pressure gradients across the villi due to elevated venous pressure linked to increased abdominal/intra-thoracic pressure, has been shown to exist, further inhibiting CSF drainage. Anatomical abnormalities in the venous sinus are often seen on neuroimaging in IIH, including transverse sinus narrowing, further compromising drainage. As with the arachnoid villi, it is debated as to whether the venous pressure and anatomical abnormalities are the consequence of raised ICP, not the cause. It has been shown that these abnormalities can reverse, albeit not always, following lowering of the ICP suggesting the latter is more likely [14,15,16]. It is currently unknown what role CSF drainage through the ‘glymphatic pathway’ may play in IIH [17].

In the adult population, androgen levels have been found to be elevated in IIH patients. Androgen receptors have been found in the choroid plexus, providing an interesting possible pathophysiological mechanism of altered CSF dynamics and raised ICP in this cohort [18].

In light of the clear differences in patient characteristics/risk factors in the pre-pubertal age group compared to the early and late post-pubertal adolescent age ranges, it is suggested the underlying pathophysiology is different in these different cohorts. This is supported by Margeta et al. who demonstrated significantly lower levels of CSF protein levels, in pre-pubertal IIH patients when compared to post-pubertal patients [19]. It is proposed that the lower CSF protein reflects an overproduction of CSF rather than reduced drainage.

At a systemic level, a recent paper by Westgate et al. elegantly investigated the underlying metabolic state of adult IIH patients. In this paper, it was shown that the elevated insulin and leptin-resistance seen in adult IIH patients was in excess of what would be expected from the obesity. The metabolic and transcriptional behaviour of adipose cells was investigated and found to be predisposed to liposynthesis indicating obesity may be a feature of the metabolic syndrome of IIH, and not a casual factor [20].

## 5. Clinical Features

### 5.1. Symptoms

Paediatric patients with IIH commonly present in one of two ways; either with symptoms of raised ICP and papilloedema, or through an incidental finding of papilloedema on a routine check-in an otherwise asymptomatic child. Rarely children with IIH may present with symptoms of raised ICP without any papilloedema [4].

On presentation, a comprehensive history should be taken from the patient or parent/carer and should include the presence of symptoms consistent with raised ICP. These typically include postural, diurnal headaches associated with nausea or vomiting, though intermittent headaches with migrainous features may also occur. Headaches, although being the most common presenting feature in children in general, are more commonly reported by older children. Other typical symptoms include transient visual obscurations, which may be brought on by Valsalva maneuvers and are seen less often in children than in adults (2–53% vs. 72%). Pulsatile tinnitus and binocular horizontal diplopia may also occur—strabismus due to a falsely localizing sixth cranial nerve palsy is more common in children, seen in up to 50% compared to up to 20% in adults [21]. In much younger children, symptoms may manifest as irritability, apathy, and somnolence. Atypical symptoms may include dizziness, ataxia, paraesthesias, and seizures [6]. A history for underlying endocrine conditions and possible iatrogenic causes of IIH (e.g., tetracyclines, Vitamin A derivatives, withdraws from chronic steroid use) should also be sought [22].

### 5.2. Signs

**Figure 1:**
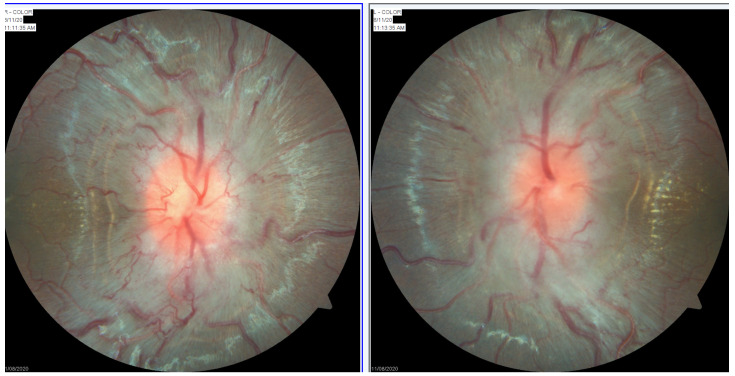
Colour optic nerve photographs showing Paton’s lines and exudates bilaterally.

**Figure 2:**
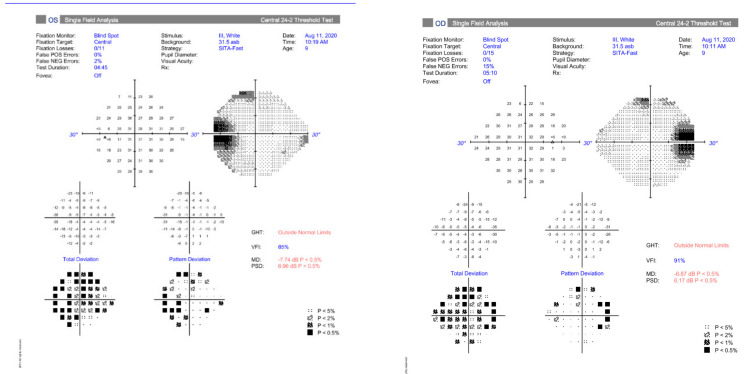
Humphrey 24-2 visual field test demonstrating bilaterally enlarged blind spots.

It is important to differentiate suspected papilloedema from pseudopailloedema to avoid potentially harmful over-diagnosis, over-investigation, and over-treatment [23]. A neuro-ophthalmic examination should include careful evaluation of optic nerve function and structure. Optic nerve head oedema is the most common sign present in paediatric IIH patients, and making the diagnosis in the absence of disc swelling, although possible, should be made with caution [4]. The clinical examination requires observation of the optic nerve margins, spontaneous venous pulsations, signs of venous congestion, haemorrhages, exudates, cotton wool spots, and Paton’s lines (Figure 1). Evaluation of optic nerve function includes visual acuity, pupil evaluation to assess for briskness of pupil constriction and for a relative afferent pupillary defect, colour vision, and visual fields – either Humphrey visual field testing, or Goldman perimetry in younger patients (Figure 2). Ocular motility assessment should not be foregone due to the lack of reported diplopia in children as the presence of a unilateral or bilateral 6th nerve palsy is frequent [6].

Any papilloedema should be graded using the Frisen scale, based on the extent of swelling and the level of obscuration of major vessels at the optic nerve margin or centre. The reliability and usefulness of grading papilloedema using the Frisen scale has been questioned. However, in the paediatric population, acquiring disc photos and optical coherence topography (OCT) imaging may not be possible due to age and compliance. In these situations, documenting the severity of optic nerve oedema using the Frisen scale is very useful for assessing changes in severity over time.

The relationship between optic disc oedema and magnitude of raised ICP is variable and on occasion, IIH may be present without papilloedema. Furthermore, in patients with optic atrophy, typical optic nerve swelling does not occur, and emphasis should be placed on detecting deterioration of the optic nerve function when assessing for elevated ICP.

**Figure 3:**
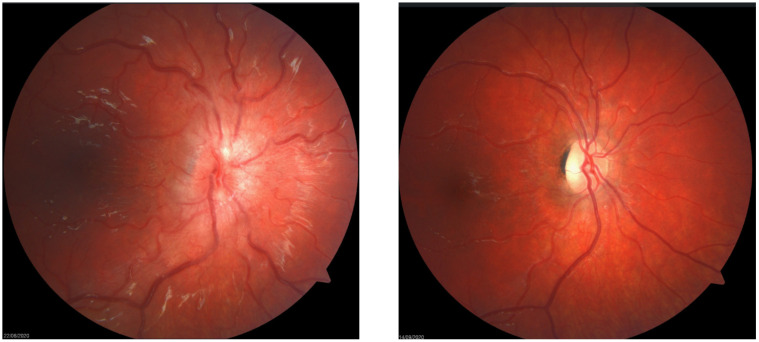
Showing Frisen scale Grade 4 Papilloedema on the left. Same disc following resolution of papilloedema resulting in optic atrophy and pallor resulting in permanent sight loss.

The major mechanism behind disc swelling in patients with raised ICP is due to elevated optic nerve sheath pressure resulting in axoplasmic stenosis and pre-laminal nerve head swelling. Secondary compression of nerve head venules results in leakage and the accumulation of extracellular fluid [24]. In extreme cases, sub-retinal fluid may track to the macula resulting in macula oedema [23]. Therefore reduced visual acuity occurs from direct optic nerve dysfunction with or without secondary macula oedema. Reduced visual acuity can be permanent if papilloedema results in ganglion cell loss and optic atrophy as shown in Figure 3. The pattern of visual field loss seen reflects the respective pathology, with macula involvement resulting in central visual loss, and optic nerve dysfunction more commonly manifesting as an enlarged blind spot, arcuate field loss, or constricted fields [25].

## 6. Work-Up and Diagnosis

### Examination and Investigations

A comprehensive history assessing for symptoms of raised ICP and possible secondary causes of intracranial hypertension should be taken. Following this optic nerve assessment should be conducted and the findings correlated against the additional investigations which should be performed.

OCT of the optic nerve is valuable to document baseline retinal nerve fibre thickness and monitor for changes over time. This is true despite the lack of normative values for nerve fibre layer thickness available for children. In eyes with papilloedema, OCT shows increased peripapillary retinal nerve fibre layer thickness with an inward deflection of the peri-papillary Bruch’s membrane layer and a smooth contour of the peripapillary subretinal space [22].

Fluorescein angiography has been shown to help differentiate between papilloedema and optic nerve head drusen [26]. Confocal scanning laser retinal tomography can quantify disc elevation above the retinal surface, showing the elevation to be greater than and extending beyond the disc margin in true papilloedema [27].

**Figure 4:**
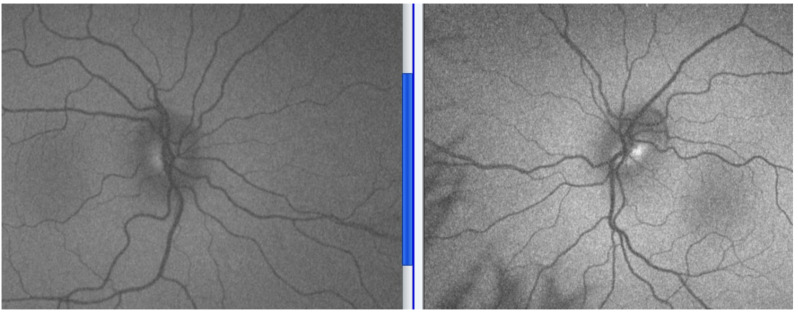
Optos fundus autofluorescence; optic disc drusen show as autofluorescence.

Ultrasound (B-scan), enhanced depth imaging OCT (EDI-OCT), and autofluorescence fundus photography can aid the identification of buried optic disc drusen, a common cause of pseudopapilloedema and misdiagnosis (Figure 4) [28]. Previous investigators have found that OCT may be less reliable in distinguishing mild papilloedema from pseudopapilloedema or buried disc drusen, with differences between RNFL thickness in these groups not being statistically significant. OCT did not always detect focal hyperreflective masses typical of disc drusen in many eyes with buried drusen; furthermore, focal hyperreflective areas were present in many eyes with papilloedema—showcasing that B-scan ultrasonography still remained more suited to distinguishing mild papilloedema from buried disc drusen [29,30]. It is important to appreciate that optic disc drusen can of course co-exist with more sinister pathology.

**Figure 5:**
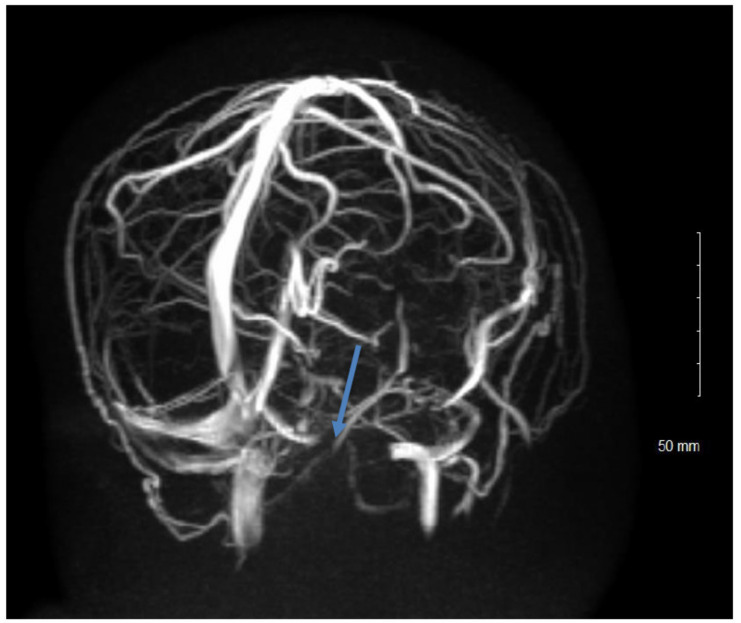
Magnetic resonance venography with blue arrow demonstrating transverse venous sinus stenosis.

Neuroimaging (MRI of the brain and orbits with and without contrast and MRV) is required to rule out other causes of raised intracranial pressure such as space occupying lesions and hydrocephalus, as well as being vital before lumbar puncture to avoid the chance of brainstem herniation. According to the revised diagnostic criteria, neuroimaging must be within normal limits except for signs of raised intracranial pressure [4]. If MRI is unavailable or contraindicated, CT with contrast can be obtained—however, this is less sensitive for lesions such as venous sinus thrombosis or stenosis—and the use of CT is generally not preferred due to the risks of radiation in children. MRI findings in paediatric patients with IIH secondary to raised ICP are similar to in adults and include protrusion of the optic nerve head into the globe, enhancement of the optic nerve head, tortuosity of the intraorbital optic nerve, increased perioptic CSF, flattening of the posterior sclera, empty sella, cerebellar tonsillar descent, meningoceles and enlargement of Meckel’s cave. Scleral flattening, transverse sinus stenosis, and sella changes were reported at lower frequencies in pre-pubertal children than adolescents [31]. Cerebral venous sinus thrombosis is a rare but important cause of ICP elevation in children hence magnetic resonance imaging venogram (MRV) should be obtained for all cases of suspected IIH [31,32]. MRV showing stenosis of the transverse venous sinuses has a high sensitivity and specificity for the diagnosis of IIH (Figure 5). CT venography is reportedly equivalent to MRV to diagnose this but should be undertaken with caution regards the radiation exposure risk which should be weighed up against the need for sedation for an MRI [33,34].

Following neuroimaging, a lumbar puncture should be undertaken – firstly to ensure CSF composition is within normal limits (excluding other causes of raised ICP) as well as to measure opening pressure. It is recommended this be performed in the lateral decubitus position with legs in flexion. Normal limits for ICP are different for the paediatric population; the definition of raised ICP in paediatric patients has been revised [4,5,35]. It is also important to bear in mind the effects of sedation (leading to a falsely positive high) on opening pressures due to hypercarbia during anaesthesia, as many children require sedation to undergo an LP.

Definite IIH would be diagnosed in a patient with bilateral optic nerve swelling and a normal neurological examination (except for cranial nerve abnormalities), normal neuroimaging (except signs associated with raised intracranial pressure)—i.e., no intracranial masses or hydrocephalus, normal CSF composition, and elevated LP pressure. Probable IIH is defined if the LP pressure is lower than what would be expected for a definite diagnosis [4].

At our institution, a large tertiary centre in the East of England, a referral pathway is in place for bilateral optic nerve oedema suspicious of IIH. The referring hospital carries out a history, documents weight, height, BMI, medications, Tanner/pubertal status, and basic blood profiles (full blood count, urea & electrolytes, liver function, haemantinics, glucose, thyroid function, vitamin D level, and lipid profile) alongside neuroimaging (MRI to include orbits, optic nerves, and pituitary fossa and MRV to rule out venous sinus thrombosis) if possible. These are sent with the referral alongside detailed referring ophthalmology reports. The referrals are triaged by paediatric neurology and paediatric neuro-ophthalmology consultants. Frisen grade 1–2 papilloedema with intact vision (or “unable to grade” referrals) are seen within 2–4 weeks of referral in the multi-disciplinary IIH clinic, with a decision on LP and CSF infusion study made [36]. Frisen grade 3–5 papilloedema or referrals for patients with compromised vision are discussed urgently with the on-call team for urgent infusion studies or urgent lumbar punctures. Lumbar CSF pressures, steady state assessments, and infusion studies are performed. Steady state assessments, averaging CSF pressure over 20 min can be more reliable than a single opening pressure measurement. CSF infusion studies, though not a standard of care, are less invasive than ICP monitoring and can be valuable in borderline cases (i.e., where steady state value is between 15 mmHg (=20 cm H_2_O, indicating normal CSF) and 20 mmHg (=27 cm H_2_0, indicating high pressure) or in the absence of papilloedema. These methods are valuable considering the dynamic nature of ICP—i.e., acknowledging that a “normal” recorded ICP at a single timepoint may not be a wholly representative value. Fundamentally, a structured, multi-disciplinary approach allows for optimal work-up and care ([37], Figure 6).

## 7. Management and Monitoring

Management should be led by a multi-disciplinary team (usually composed of a paediatrician, paediatric neurologist, ophthalmologist, orthoptist, nutritionist, and neurosurgeon) [38]. The aim of treatment is to alleviate symptoms and prevent complications – namely visual loss. Treatment strategies in the paediatric population are based on retrospective studies and mostly follow approaches used in adults; no randomised controlled trial studies in children are available to date [39]. Treatment is thereby influenced by the level of vision loss and severity of symptoms. It has been suggested that for younger children where follow-up with perimetry is more difficult, treatment could be more aggressive—as should children with severe papilloedema at presentation due to worse visual outcomes [40,41].

### 7.1. Conservative Measures

In terms of conservative measures—any medications known to advance IIH should be stopped if possible. Counselling on weight loss (with a recommendation for 10% loss of the child’s weight) should be provided [5,42,43]. It has been suggested that bariatric surgery can be considered in morbidly obese children with IIH with unsuccessful weight loss trials, should the obesity be associated with other complications such as sleep apnoea or diabetes [44]. A recent 5-year randomised control trial showed sustained disease remission in subjects who had bariatric surgery (compared to a community weight management intervention) in overweight adult women patients with active IIH [45].

Observation is a reasonable option for patients with normal vision and mild papilloedema [5]. Although serial lumbar punctures have been proposed as an option for adult patients, it is less preferred in a paediatric population, being painful, poorly tolerated, and having short-lived effects—though occasionally the diagnostic LP also has a therapeutic effect with potentially no requirement for any further treatment [40].

### 7.2. Medications

The mainstay of medical management includes the use of acetazolamide, topiramate, and corticosteroids.

Acetazolamide: Acetazolamide works by decreasing the production of CSF from the choroid plexus by inhibiting carbonic anhydrase and has shown effectiveness in some small prospective exclusively paediatric studies [44]. Although there is no RCT in the paediatric population, the Idiopathic Intracranial Hypertension Treatment Trial can offer valuable lessons especially with regard to postpubertal patients who appear to behave similarly to adults [46]. This trial evaluated acetazolamide plus weight reduction and/or low sodium diet use as consistent with papilloedema improvement as well as visual acuity and vision-related quality of life. The standard dosing regimen for children is 15–25 mg/kg/day. Adverse effects include nausea, vomiting, and paraestheisas; it is important electrolytes (i.e., potassium and bicarbonate) are monitored for at least every three months as metabolic acidosis may occur insidiously.

Topiramate: Topiramate, though relatively new in treating IIH, has been safely used for years as an anti-epileptic in paediatric populations and has secondary carbonic anhydrase activity. It can additionally suppress appetite leading to weight loss in patients and has headache prophylaxis properties. It is dosed at 1.5–3 mg/kg/day in 2 divided doses and can be titrated up slowly over weeks to 200 mg/day [5].

Furosemide: Furosemide, a loop diuretic, tends to be used if acetazolamide is not tolerated or as adjunctive therapy, at a dose of 0.3–0.6 mg/kg/day. It is suggested that combining the two can produce additive results [47].

Steroids: Although the use of chronic steroids are avoided due to their side effect profile as well as rebound-phenomenon when withdrawn, IV methylprednisolone at a dose of 15 mg/kg can be used in situations of acute, severe visual loss whilst awaiting surgery [48].

### 7.3. Surgical Options

Should medical treatment not provide a satisfactory ICP reduction (or should a more urgent/immediate reduction in ICP be required if visual field loss is severe), surgical options are considered. Various surgical options for IIH have been described. The Surgical Idiopathic Intracranial Hypertension Treatment Trial (SIGHT), set up to compare various surgical and medical options, was unfortunately closed due to the feasibility of recruitment and choice of procedure, making evidence-based surgical decisions, even in the adult population, challenging [47,49].

Shunting: This can include the placement of a ventriculoperitoneal or lumboperitoneal shunt [31]. Complications of CSF shunting include shunt obstruction, shunt infections, lumbar radiculopathy, tonsillar herniation, need for shunt revision, and CSF leak—and children may have a higher risk of developing complications possibly due to increased growth, or size of the shunt tubing in their thecal sac [5].

Optic nerve sheath fenestration: Optic nerve sheath fenestration (ONSF) has not shown superiority to shunt placement however it may be considered should a patient be unsuitable for more invasive procedures, especially if vision loss is the major issue and if a headache is not a significant feature. Paediatric case series suggest it as a safe, effective way of stabilising visual function when measuring resolution of papilloedema, visual acuity, and fields stabilisation/improvement, highlighting an efficacy similar to that in adults [50,51]. Optic disc swelling may improve bilaterally despite just a unilateral procedure though the mechanism is not clear, it is postulated this is due to CSF filtration through the posterior orbit and optic chiasm [22]. However, it is unclear how long the effect of ONSF lasts—3 of 25 children undergoing ONSF in the literature experienced post-operative visual worsening and further procedures may be necessary [5].

Venous sinus stenting: Venous sinus stenting is an alternative option in patients with refractory IIH with the aim of reducing venous pressures by improving cerebral venous outflow and allowing more efficient resorption of CSF through subarachnoid granulations to the dural venous sinuses. A recent review of 28,794 new IIH cases (including paediatric patients) showed stenting is less commonly performed than shunting; in addition, long-term safety data in the paediatric population is limited and consideration must be given to the fact that presently there is no way to remove such a stent once placed [50,51,52].

Headaches should generally resolve as ICP reduces; however conventional analgesia can be used if required. Monitoring of visual function can be challenging in a paediatric population. It has been suggested that an experienced examiner can measure colour vision by age four and map visual fields in those aged five and over [33]. Visual field testing (with associated abnormalities being an enlarged blind spot, arcuate defects, infero-nasal field loss, global constriction, generalized depression, and cecocentral scotomas) is more sensitive than visual acuity - but may not be possible for younger patients [27]. Decreased visual acuity and reduced colour vision are easier to elicit but are late signs of papilloedema. Evaluation of the disc appearance is subjective and can lag behind visual changes. Obtaining VEPs may be technically difficult—and is insensitive to visual loss in IIH [22,43].

OCT shows to be an encouraging investigation to use not only to aid diagnosis in children unable to perform field tests, but also as a surrogate monitoring marker correlating with ICP. Peri-papillary retinal nerve fibre layer (pRNFL) thickness is increased in patients with IIH—however caution must be exercised when using pRNFL to monitor IIH as a decrease may relate to axonal death with severe or chronic papilloedema—not just improving papilloedema. This can be differentiated by correlating the decreasing pRNFL with deterioration in visual function and looking concurrently at the macular ganglion cell layer-inner plexiform layer (GCL-IPL) thickness—if this is reduced alongside pRNFL thickness reduction, it is more likely to represent worsening optic neuropathy from severe or chronic papilloedema—however, this must be assessed over time as there is a 2-week time lag between optic nerve damage and macular GCL-IPL layer thickness reduction [22].

The prognosis for paediatric IIH patients is generally good—most children with mild to moderate field defects will have total resolution of their symptoms with a resolution of papilloedema in 4–5 months [53]. The recurrence rate is between 6–22% and anecdotally occurs in overweight adolescents who initially lose then subsequently regain the weight. Permanent visual function loss (regardless of treatment) has been previously reported in children with reduced visual acuity in up to 10% and permanent visual field defects in up to 17% of children [39]. The risk of permanent loss is highest in severe or long-standing disc swelling when ganglion cell loss can occur, and therefore permanent defects will remain even when the oedema resolves. It is therefore of the upmost importance to ensure all reasonable measures are taken for accurate work-up, diagnosis, management, and monitoring.

## Data Availability

Not applicable.
